# Iron-polyphenol complexes cause blackening upon grinding *Hermetia illucens* (black soldier fly) larvae

**DOI:** 10.1038/s41598-019-38923-x

**Published:** 2019-02-27

**Authors:** Renske H. Janssen, Greta Canelli, Mark G. Sanders, Edwin J. Bakx, Catriona M. M. Lakemond, Vincenzo Fogliano, Jean-Paul Vincken

**Affiliations:** 1 Food Quality and Design, Wageningen University and Research, PO Box 17, 6700 AA Wageningen, The Netherlands; 2 Laboratory of Food Chemistry, Wageningen University and Research, PO Box 17, 6700 AA Wageningen, The Netherlands

## Abstract

Insects are a promising alternative protein source. One of the bottlenecks in applying insects in food is the fast darkening initiated during grinding. Besides enzymatic browning, non-enzymatic factors can cause off-colour formation, which differs between species. This study investigates the impact of iron, phenoloxidase, and polyphenols on off-colour formation in insect larvae. *Hermetia illucens* showed a blackish colour, whereas *Tenebrio molitor* turned brown and *Alphitobius diaperinus* remained the lightest. This off-colour formation appeared correlated with the iron content in the larvae, which was 61 ± 9.71, 54 ± 1.72 and 221 ± 6.07 mg/kg dw for *T*. *molitor*, *A*. *diaperinus* and *H*. *illucens*, respectively. In model systems, the formation of iron-L-3,4-dihydroxyphenylalanine (L-DOPA) bis- and tris-complexes were evidenced by direct injection into ESI-TOF-MS, based on their charges combined with iron isotope patterns. The reversibility of the binding of iron to phenolics, and thereby loss of blackening, was confirmed by EDTA addition. Besides complex formation, oxidation of L-DOPA by redox reactions with iron occurred mainly at low pH, whereas auto-oxidation of L-DOPA mainly occurred at pH 10. Tyrosinase (i.e. phenoloxidase) activity did not change complex formation. The similarity in off-colour formation between the model system and insects indicated an important role for iron-phenolic complexation in blackening.

## Introduction

Insects are nowadays investigated as alternative protein source to meet the growing protein demand in the future^1^. Insects can be a protein source for feed because of their well-balanced amino acid profile and the sustainability of their rearing^2,3^. Larvae from *T*. *molitor* (yellow mealworm) and *A*. *diaperinus* (lesser mealworm) are under investigation, as they are reared for food and feed in The Netherlands; while *H*. *illucens* larvae (black soldier fly) are mainly used for feed. To use insects as ingredient in food and feed, grinding is often necessary. Fast off-colour formation occurs during this treatment, which is partly caused by enzymatic browning catalysed by phenoloxidase, also called tyrosinase^4^. Nevertheless, the oxidative enzyme activity of the different species cannot explain the differences in off-colour formation of *Hermetia illucens* compared to that of *Tenebrio molitor* and *Alphitobius diaperinus*. *H*. *illucens* showed a marked black colour after grinding, whereas *T*. *molitor* was deep brown, and *A*. *diaperinus* yellowish. The highest enzyme activity was found for *T*. *molitor*, then *A*. *diaperinus* and the lowest activity was found for *H*. *illucens*, based on oxygen consumption measurements with L-3,4-dihydroxyphenylalanine (L-DOPA) as substrate^4^.

Multiple insect species have been shown to contain a high iron content^2,5^. Minerals like iron are important as micronutrient source in food and feed. Iron deficiency is the most common nutritional disorder in the world, according to The World Health Organization^6^. Nevertheless, iron ions are reactive and are known to induce off-colour formation in fortified foods, often caused by their interaction with polyphenols^7,8^.

In insects, iron is both an essential nutrient and a strong toxin. Insects contain transferrin proteins to transport iron in serum and ferritin proteins to store it. Other proteins that contain iron are haemoglobin and myoglobin, which both transport and store oxygen^9^. Transferrin binds only one ferric iron (Fe^3+^). Ferritin can bind multiple ferrous iron (Fe^2+^) which is then converted into the ferric form and stored as such in the oxoferrihydrite core^9^.

Polyphenols in insects are substrates for oxidative enzymes contributing to the immune response, wound healing and sclerotization of the cuticle^10,11^. Polyphenols in insects usually reside in the haemolymph in the form of L-tyrosine^12^, besides small amounts of L-tyrosine derivatives like L-dopamine and L-DOPA, often in phosphorylated or glycosylated form^10^. These polyphenols are prone to oxidation by endogenous enzymes or iron, although the latter has never been studied in insects. Iron oxidation is mediated by redox reactions, in which dihydroxyphenolic compounds can serve as reducing agents^7^, as shown in Eq. (1)^13^. The product DOPAquinone can react further non-enzymatically to form melanins or crosslinks with proteins^14^.

1 $$2{{Fe}}^{3+}+{DOPA}\to 2{{Fe}}^{2+}+{DOP}\,{Aquinone}+2{{H}}^{+}$$

Polyphenols with low redox potentials are most easily oxidized by ferric iron, causing off-colour^7^. Ferrous iron needs to be converted into ferric iron by e.g. oxygen to form a complex with, and/or oxidize, these phenolics^7,15^.

Besides oxidation, *ortho*-hydroxy polyphenols can also form complexes with minerals, e.g. iron^7^. For example, L-DOPA has been described to form complexes with ferric iron (Fe^3+^) in marine mussel threads. Mussel foot proteins contain large quantity of L-DOPA and in presence of iron, these L-DOPA residues react by intermolecular crosslinking^16,17^. Black colour formation is observed when the L-DOPA residues in the proteins chelate Fe^3+^ into this intermolecular complex^16^. Ferric iron shows approximately ten times more intense colour formation with catechol compared to ferrous iron (Fe^2+^), underlining the importance of iron to be in the oxidized form^7^.

The chelating ability increases with increasing pH, as a larger proportion of the hydroxyl groups resides in the dissociated form^15^. Below pH 5, iron is complexed with only one L-DOPA, between 5.6–9.1 the bis form is dominant, and above 9.1, the tris form^18^.

The aim of this research was to understand the differences in colour formation upon grinding between *Hermetia illucens* in comparison to *T*. *molitor* and *A*. *diaperinus* with emphasis on the impact of tyrosinase, phenolics and minerals. To investigate this, a model system was developed, containing L-DOPA, iron and tyrosinase, all parameters that potentially play a role in off-colour formation upon insect processing. It was hypothesized that, besides enzymatic browning, iron-DOPA complexes play a role in off-colour formation in insects, and explain the blackish colour in *H*. *illucens*.

## Results and Discussion

### Off-colour formation upon grinding of insect larvae under different conditions

**Figure 1:**
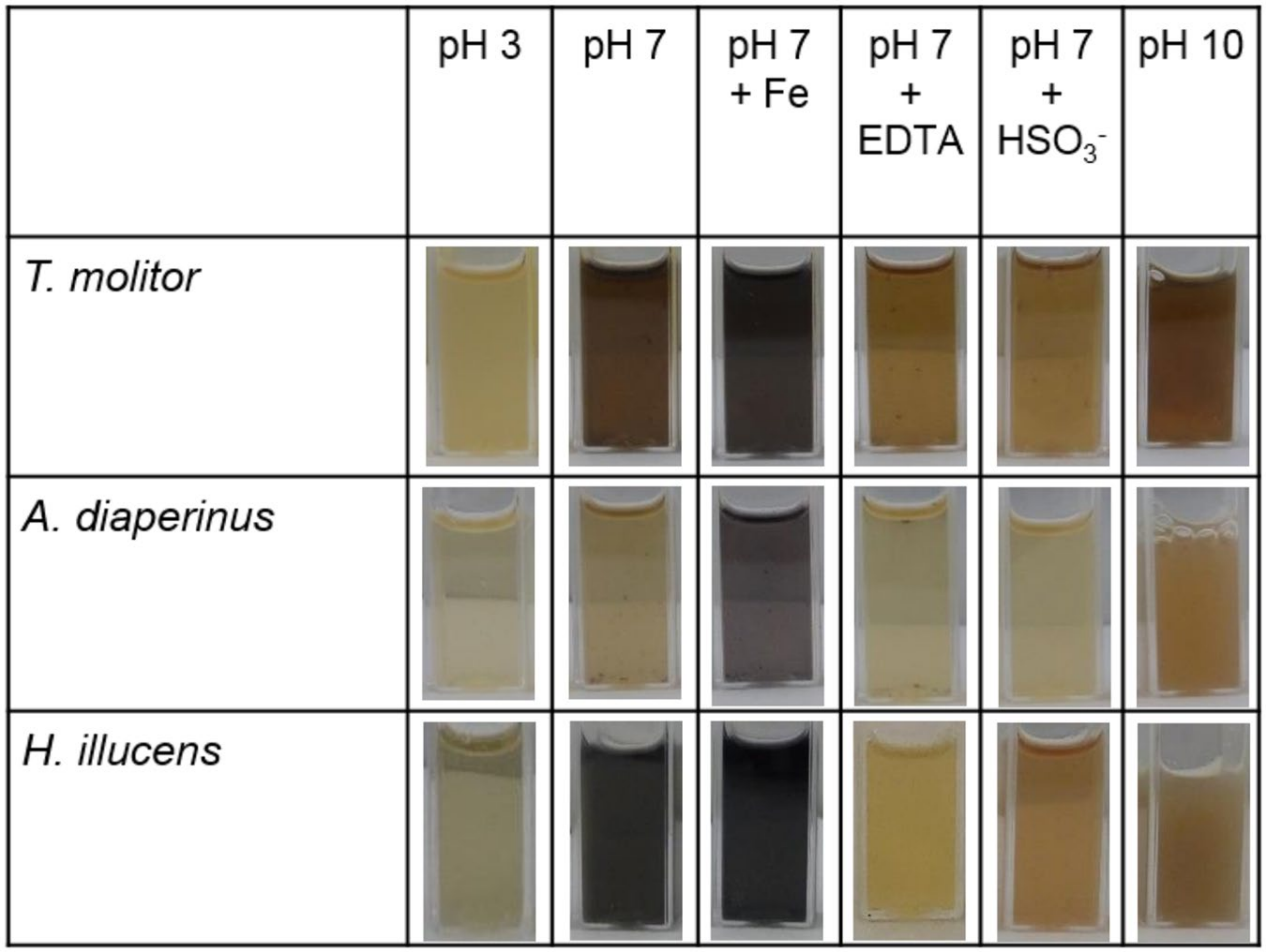
Off-colour formation at pH 3, 7 and 10 of extracts from *Tenebrio molitor*, *Alphitobius diaperinus* and *Hermetia illucens* 1 h after grinding. At pH 7 sodium bisulphite (1 mM), FeCl_3_ (3 mM) and EDTA (1 mM) were added. Extra EDTA was added to *H*. *illucens* to obtain a lighter colour (∼4 mM).

Off-colour formation was observed in extracts from larvae of three insect species at different conditions (Fig. 1). Dark brown or black colour was observed at pH 7, whereas at pH 3 the colour formation was limited for all species, probably because phenoloxidase was inactive at low pH^4^. At pH 10, the dark brownish colour was attributed to auto-oxidation of polyphenols, which is known to be enhanced at high pH^17^.

The extracts from the various species responded differently to the various conditions, especially at neutral pH. The least off-colour formation was found for *A*. *diaperinus*, whereas *T*. *molitor* showed darker brown colour and *H*. *illucens* black colour. The colour at neutral pH is relevant for future application.

To investigate the effect of phenoloxidase on colour formation of the different species, sulphite was added. Sulphite is known to inhibit phenoloxidase irreversibly in a time-dependent manner^19^. Besides inhibition of phenoloxidase, sulphite can act as reducing agent by reducing ferric ion back to ferrous ion and lighten the extract^7^. All extracts became lighter upon sulphite addition, indicating that phenoloxidase caused part of the off-colour formation. Nevertheless, only phenoloxidase could not explain differences between species in discolouration^4^.

To investigate the difference in off-colour formation between species at pH 7, extra iron (FeCl_3_) was added during grinding. The colour of *T*. *molitor* and *A*. *diaperinus* extracts darkened upon iron addition and became more similar (blackish) to the colour of *H*. *illucens* without iron addition, indicating a role for iron in colour change. The addition of the chelating agent EDTA resulted in lighter colour of all insect extracts at pH 7, and provided further support for this theory (see Fig. 1)^20,21^. As part of the off-colour could be removed upon EDTA addition, it was hypothesized that iron was chelated by L-DOPA and not by quinones that had reacted further into coloured products.

### Mineral analysis of insect larvae

The mineral composition of *T*. *molitor*, *A*. *diaperinus* and *H*. *illucens* was investigated in order to correlate this to the off-colour formation observed in insect extracts (Table 1). Both *T*. *molitor* and *A*. *diaperinus* showed comparable mineral composition, whereas *H*. *illucens* gave significant differences in the content of the minerals calcium, iron, manganese, sodium and zinc. The iron content of *H*. *illucens* was four times higher than that of the other species. Interestingly, the darkest colour was formed with *H*. *illucens*, despite the fact that it had the lowest tyrosinase activity^4^. Differences in iron content, in particular, might explain differences in colour formation between species, as iron is known for off-colour formation in e.g. fortified products^8^ and iron addition was observed to darken the extracts in Fig. 1. Besides, the colour formation depends on oxidative state of the iron^7^.

**Table 1:** Mineral composition of *Tenebrio molitor*, *Alphitobius diaperinus* and *Hermetia illucens* larvae based on dry matter (n = 3, mean ± S.D.).

Larvae	Ca [g/kg]	Cu [mg/kg]	Fe [mg/kg]	K [g/kg]	Mg [g/kg]	Mn [mg/kg]	Na [mg/kg]	P [g/kg]	S [g/kg]	Zn [mg/kg]
*T. molitor*	1 ± 0.3	13.4 ± 0.5	61.0 ± 9.7	9.7 ± 0.4	2.2 ± 0.3	10.8 ± 2.1	1737.3 ± 106.0	7.6 ± 0.7	3.5 ± 0.2	111.0 ± 13.1
*A. diaperinus*	0.5 ± 0.0	21.9 ± 0.4	53.5 ± 1.7	10.0 ± 0.2	1.3 ± 0.0	5.4 ± 0.3	2270 ± 116.8	8.4 ± 0.4	4.3 ± 0.2	140.6 ± 4.3
*H. illucens*	26.6 ± 1.8	13.2 ± 1.0	220.7 ± 6.1	8.9 ± 0.3	2.0 ± 0.2	338.0 ± 49.1	673.7 ± 25.9	6.9 ± 0.3	3.1 ± 0.0	93.0 ± 3.1

### Phenolics involved in off-colour formation in larvae from *T*. *molitor*, *A*. *diaperinus* and *H*. *illucens*

To investigate which phenolics play a role in off-colour formation of insects, darkened and light extracts were investigated (Table 2). Decreases in phenolics of at least two-fold in the brown extract compared to the light extract were considered diagnostic for participation of these phenolics in off-colour formation. Furthermore, only abundant phenolics with an area of at least 10^6^ in the light extracts were taken into account.

**Table 2:** Phenolics involved in browning of *Tenebrio molitor*, *Alphitobius diaperinus* and *Hermetia illucens* during grinding and their decrease upon darkening, per kg dry matter (n = 3, ±SD). The molar ratio L-tyrosine was included as well.

	Extract	*T. molitor*	*A. diaperinus*	*H. illucens*
L-tyrosine (mg/kg)	Light	2557 ± 190	1912 ± 263	2674 ± 253
	Dark	651 ± 140	205 ± 8	105 ± 13
L-DOPA (mg/kg)	Light	40 ± 7	27 ± 4	21 ± 2
	Dark	36 ± 1	10 ± 4	3 ± 1
	Molar ratio L-tyrosine: Fe	13	11	4

Table 2 shows that L-tyrosine had a high initial concentration and the largest decrease upon darkening for all three species. L-tyrosine is known to be present in insect haemolymph^12^. Upon browning, the largest decrease of tyrosine was 25 times for *H*. *illucens*, whereas a nine and four times decrease was found for *A*. *diaperinus* and *T*. *molitor*, respectively.

L-tyrosine itself cannot form a complex with iron, as two *ortho*-hydroxyl groups are necessary. L-tyrosine should therefore be first hydroxylated into L-DOPA^7^. The concentration of L-DOPA (mg/kg) was found to be low in both dark and light insect extracts compared to L-tyrosine. Likely, L-DOPA, formed from L-tyrosine, was either oxidized into quinones upon reaction with endogenous phenoloxidase, or complexed with minerals. The most important precursor in darkening substrates was L-tyrosine. The ratio of reactive phenolics, in this case L-tyrosine, and iron was four times lower for *H*. *illucens* compared to *A*. *diaperinus* and *T*. *molitor*. Given the observation that the L-tyrosine concentration in light extracts is similar for the three different insect extracts, the differences in iron content might explain the difference in off-colour formation between the species.

### Factors affecting colour formation with diphenolic structures

#### Ferric ion interacts with diphenolic structures causing colour formation

To investigate the impact of phenolics and minerals on colour formation, a model system was set up. As *ortho*-hydroxyl groups are necessary for complex formation^7^, L-DOPA was used in the model system instead of its precursor L-tyrosine.

First, L-DOPA was combined with one mineral at a time. Colour formation was only observed with iron and not with any of the other minerals tested (Supplementary Fig. S1), of which the content that different among the three insect species as shown before in Table 1.

To investigate the effect of oxygen on colour formation, ferric and ferrous iron were combined with L-DOPA with and without reduced oxygen levels. Ferrous iron only formed colour in presence of oxygen, whereas ferric iron also gave colour without oxygen (Supplementary Fig. S2). Thus, oxygen was necessary to oxidize iron into the ferric form to be able to react with L-DOPA. This was confirmed by mass spectrometric analysis of L-DOPA-iron complexes (Supplementary Fig. S3, see further). Chloride was used as counter ion, as this counter ion did not influence oxidation as shown before^22^. Therefore, ferric iron chloride (FeCl_3_) was used in subsequent experiments.

#### Effect of pH on colour formation

**Figure 2:**
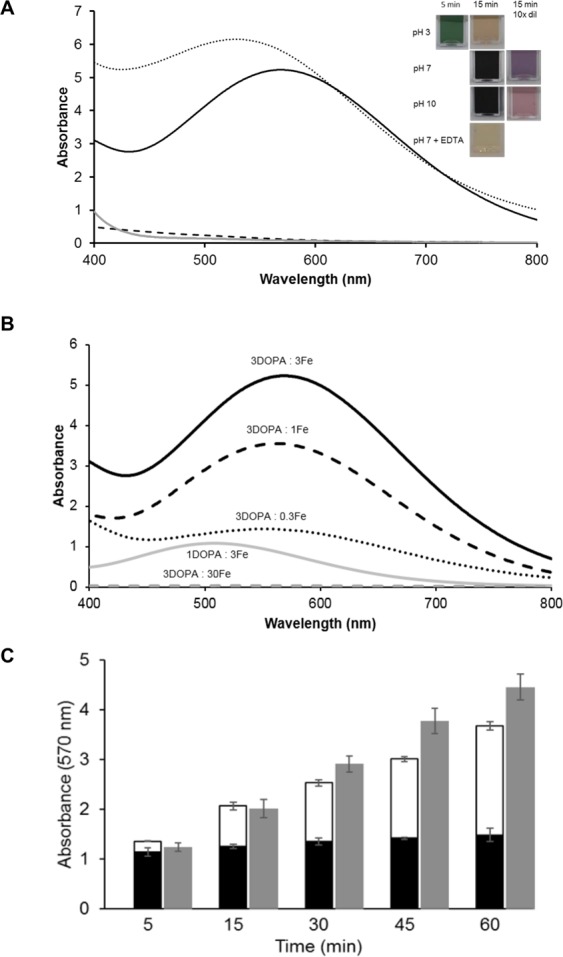
Panel (A) Absorbance spectra at pH 3 (dashed line), 7 (solid black line), 7 + EDTA (solid grey line) and 10 (dotted line) on absorbance of equimolar (3 mM) L-DOPA and FeCl_3_ with hypsochromic shift from pH 7 to 10. The colours are shown at pH 3, 7 and 10, as such and 10 times diluted if necessary. The green colour at pH 3 was directly formed after addition of iron and L-DOPA and the absorbance was measured after 15 min without dilution. The sample containing EDTA was also measured without dilution. Panel (B) Absorbance of sample containing different combinations of L-DOPA + ferric iron: respectively 1 and 3 mM (grey line), 3 and 30 mM (grey dashed line), 3 and 0.3 mM (black dotted line), 3 and 1 mM (black dashed line), 3 and 3 mM (black line). Panel C: Colour formation of L-DOPA and iron for molar ratio 1:1 (black), L-DOPA with tyrosinase (white) and L-DOPA with iron and tyrosinase (grey) at 570 nm over time (n = 3).

Insect extracts showed different colour at different pH values. The colour of the model system with L-DOPA after addition of iron at different pH values is shown in Fig. 2A. Although similar black colour was formed after addition of iron at pH 7 and 10, differences are observed after dilution. The diluted model system at pH 7 had a more purple colour, whereas that at pH 10 was more reddish corresponding to a hypsochromic shift in the visible light spectrum from 570 nm at pH 7 to 520 nm at pH 10 (Fig. 2A). The absorbance peak at 570 nm for pH 7 has been reported before to correspond to an iron-L-DOPA complex^13,16^. The chelating agent EDTA lightened the colour, indicating that iron and L-DOPA interacted non-covalently with each other. Increased absorption at 520 nm was suggested to represent dopachrome formation upon auto-oxidation of L-DOPA at basic pH^19^. The least colour formation was observed at pH 3, suggesting no complex formation between L-DOPA and iron. This might be due to the competition between protons and metal ion for the L-DOPA binding site, which is more favourable for protons at low pH^20^. However, a peculiar green colour formation was observed at pH 3 a few minutes after combining L-DOPA and Fe^3+^. This colour was attributed to Fe^2+^-semiquinone complexes, where the semiquinone radical is stabilized by the aromatic ring. Fe^2+^ was formed from Fe^3+^ by redox reaction as shown in Eq. (1). Subsequently, the semiquinone reduced another equivalent of Fe^3+^ to Fe^2+^ leading to a simultaneous oxidation of the semiquinone to quinone^13^. This changed the green colour to a light brown colour, which was probably due to products obtained by e.g. oxidative coupling of quinones and phenolics^23^.

#### Effect of ratio L-DOPA: Fe^3+^ on colour

The effect of different ratios of L-DOPA and iron concentrations on colour formation were tested at pH 7. Figure 2B shows the absorbance of L-DOPA (3 mM) and different concentration of Fe^3+^ (0.3, 1, 3, 30 mM), as well as L-DOPA (1 mM) and Fe^3+^ (3 mM). Samples with excess of L-DOPA showed the highest absorbance at 570 nm, whereas excess of iron showed a shift towards 520 nm. Thus, iron excess promoted L-DOPA oxidation by redox reaction, rather than iron-phenolics complexation. This was further indicated by the substantial precipitation of material after centrifuging samples from incubations with L-DOPA (3 mM) and iron (30 mM). The precipitate probably contained insoluble products formed upon L-DOPA oxidation in presence of high amount of iron by redox reaction.

#### Effect of tyrosinase on colour formation

In insects, not only iron, but also phenoloxidase can react with phenolics. Phenoloxidase can oxidize diphenols into quinones. The effect of tyrosinase (phenoloxidase from fungal source) on colour formation with L-DOPA in presence of iron was investigated over time in a model system. The complexation of L-DOPA and iron was fast and did not significantly (*P* < 0.05) change over time at pH 7 (Fig. 2C). Oxidation of L-DOPA by tyrosinase on the other hand increased slowly over time observed at 520 nm (data not shown). A similar trend was observed at 570 nm, the absorbance where DOPA-iron complexes were observed (Fig. 2A,B), as also described in literature^13,16^. Thus, absorbance at 570 nm did not solely reflect complexation of iron and L-DOPA, but also oxidation of phenolics.

The dark colour also increased over time in presence of both tyrosinase and iron. This increase in colour formation was mainly due to oxidation, as the absorbance of L-DOPA plus tyrosinase showed a similar absorbance to the reaction with all combined. No synergy between iron and tyrosinase was found indicating that it is unlikely that iron-quinone complexes caused the colour formation. This was in accordance with the lightening of the mixture after EDTA addition, showing the reversibility of off-colour formation in insects (Fig. 1) and in the model system with EDTA and equimolar iron and L-DOPA (Fig. 2A). The colour formation at pH 7 was an interplay between L-DOPA complexation with iron (instant) and oxidation by tyrosinase (over time).

### Iron-DOPA complex formation and oxidation analysed by ESI-TOF-MS

To elucidate the structures and intensity of the complexes at different pH values, samples were measured in negative mode mass spectrometry. This was done using direct injection into the mass spectrometer to avoid losing the non-covalently bound iron-L-DOPA complexes upon chromatographic separation. Also, the total intensity of L-DOPA in positive mode was determined in this way.

The sample containing equimolar concentration of L-DOPA and iron (3 mM) at pH 7 showed the most intense colour formation in the preliminary experiments. Potential complexes with iron were found at *m/z* 386.05, 446.06, 482.03 and 643.13. These signals were not observed in the control with only L-DOPA and upon iron chelation by EDTA, indicating once more the involvement of iron in complex formation, and confirming that complex formation is reversible (Supplementary Fig. S4**)**. Moreover, at *m/z* 344.01 a large peak was observed after EDTA addition, indicating the presence of EDTA-iron complex [(EDTA-4H^+^) +Fe^3+^]^−^ upon chelation^24^.

The presence of iron in the identified complexes was confirmed by the isotope pattern, as this is specific for iron with 5.85% ^54^Fe and 91.75% ^56^Fe. Besides that, also 2.12% ^57^Fe and 0.28% ^58^Fe was present. Table 3 shows the proposed structures with the theoretical and experimental isotope abundance. The structures were proposed according to the *m/z* value, fragmentation pattern and isotope abundance in comparison to the theoretical abundance.

**Table 3:** Tentative structures of [2(DOPA-2H^+^) + Fe^3+^]^−^, [2(DOPA-2H^+^) + Fe^3+^ + 2H_2_O]^−^, [3DOPA + Fe^3+^ − 4H^+^]^−^ with corresponding experimental and theoretical relative isotopic intensity measured in negative mode.

Tentative structure	Isotopes	*m/z*		Isotope abundance (%)	
		Exp.	Theoretical	Exp.	Theoretical
	^12^C_18_H_18_N_2_O_8_^54^Fe	444.06	444.05	3.2	6.4
	^12^C_17_^13^C_1_H_18_N_2_O_8_^54^Fe	445.05	445.05	0.6	1.2
	^12^C_18_H_18_N_2_O_8_^56^Fe	446.06	446.06	100	100
	^12^C_17_^13^CH_18_N_2_O_8_^56^Fe or ^12^C_18_H_18_N_2_O_8_^57^Fe	447.06	447.04	16.0	21.8
	^12^C_16_^13^C_2_H_18_N_2_O_8_^56^Fe or ^12^C_18_H_18_N_2_O_8_^58^Fe	448.06	448.05	1.2	3.4
	^12^C_18_H_22_N_2_O_10_^54^Fe	480.03	480.07	5.5	6.4
	^12^C_17_^13^C_1_H_22_N_2_O_10_^55^Fe	481.02	481.07	1.6	1.2
	^12^C_18_H_22_N_2_O_10_^56^Fe	482.03	482.06	100	100
	^12^C_17_^13^CH_22_N_2_O_10_^56^Fe or ^12^C_18_H_22_N_2_O_10_^57^Fe	483.04	483.07	23.0	21.8
	^12^C_16_^13^C_2_H_22_N_2_O_10_^56^Fe or ^12^C_18_H_22_N_2_O_10_^58^Fe	484.03	484.07	33.7	3.9
	^12^C_27_H_29_N_3_O_12_^54^Fe	641.13	641.12	3.8	6.3
	^12^C_26_^13^C_1_H_29_N_3_O_12_^54^Fe	642.13	642.12	1.6	2.0
	^12^C_27_H_29_N_3_O_12_^56^Fe	643.13	643.11	100	100
	^12^C_26_^13^CH_29_N_3_O_12_^56^Fe or ^12^C_27_H_29_N_3_O_12_^57^Fe	644.14	644.11	26.8	33.3
	^12^C_25_^13^C_2_H_29_N_3_O_12_^56^Fe or ^12^C_27_H_29_N_3_O_12_^58^Fe	645.15	645.12	6.1	8.1

Peaks at *m/z* 446.06, 482.03 and 643.13 were tentatively annotated as respectively [2(DOPA-2H^+^) +Fe^3+^]^−^, [2(DOPA-2H^+^) +Fe^3+^ + 2H_2_O]^−^ and [3DOPA + Fe^3+^−4H^+^]^−^, as shown in Table 3 based on MS^2^ fragmentation. Supplementary Fig. S5 shows the MS^2^ spectrum of [2(DOPA-2H^+^) +Fe^3+^ + 2 H_2_O]^−^ with *m/z* 482.03, with the positions of fragmentation (leading to the daughter ions) projected on the proposed chemical structure. Upon fragmentation, two water molecules were removed (*m/z* 35.97) and a loss of [DOPA-H^+^]^−^ (*m/z* 196.07) occurred, confirming the presence of L-DOPA in the complex and not the quinones.

The peaks *m/z* 386.06 and 536.05 were also removed upon EDTA addition, and showed the specific iron isotope pattern. As *m/z* 386.06 was found in the fragmentation pattern of *m/z* 446.04 and *m/z* 482.03, this peak likely originated from in-source fragmentation. The structure of *m/z* 536.05 remains unclear, but it is also fragmented, among others, to *m/z* 446.03. Both peaks were therefore regarded as iron-phenolic complexes and they were accounted for when estimating the abundance of iron-phenolic complexes.

#### Ferric iron in complex

The complex at *m/z* 446.06 was proposed as two L-DOPA molecules chelating Fe^3+^, [2(DOPA-2H^+^) +Fe^3+^]^−^. Although the pKa of the hydroxyl groups in L-DOPA is known to be around 9–10, it is possible that suitable cations, like Fe^3+^, can displace protons at lower pH values, e.g. 5.0–8.0^25^. Therefore, it is likely that the iron interacted with the negatively charged hydroxyl groups of L-DOPA. Moreover, as the complex was detected with an overall charge of −1 in negative mode, it was suggested that two carboxyl groups were protonated.

In all identified complexes, the iron ion in the centre was likely to be in the ferric form, based on the overall charge. This is consistent with a previous report, which also described Fe^3+^-catecholate and Fe^3+^-gallate complexes^13^. As complexes with Fe^3+^ are more stable than complexes with Fe^2+^, complexes with Fe^2+^ rapidly oxidize to complexes with Fe^3+^ via auto-oxidation^13^. This was confirmed by the results in Supplement Fig. S2, showing the necessity of oxygen to convert ferrous iron into ferric iron to form colour. Also, less iron-DOPA complexes were formed with ferrous iron compared to ferric iron (Supplement Fig. S3).

Taken together, our data suggested that iron acted as a ligand-to-metal-charge-transfer (LMCT), allowing the electrons of the negatively charged hydroxyl groups to be delocalized over both ligands^13,26,27^. Thus, the enlarged electronic system caused black colour formation and UV absorbance at 570 nm.

#### Effect of pH and ratio L-DOPA: Fe on complex formation

**Figure 3:**
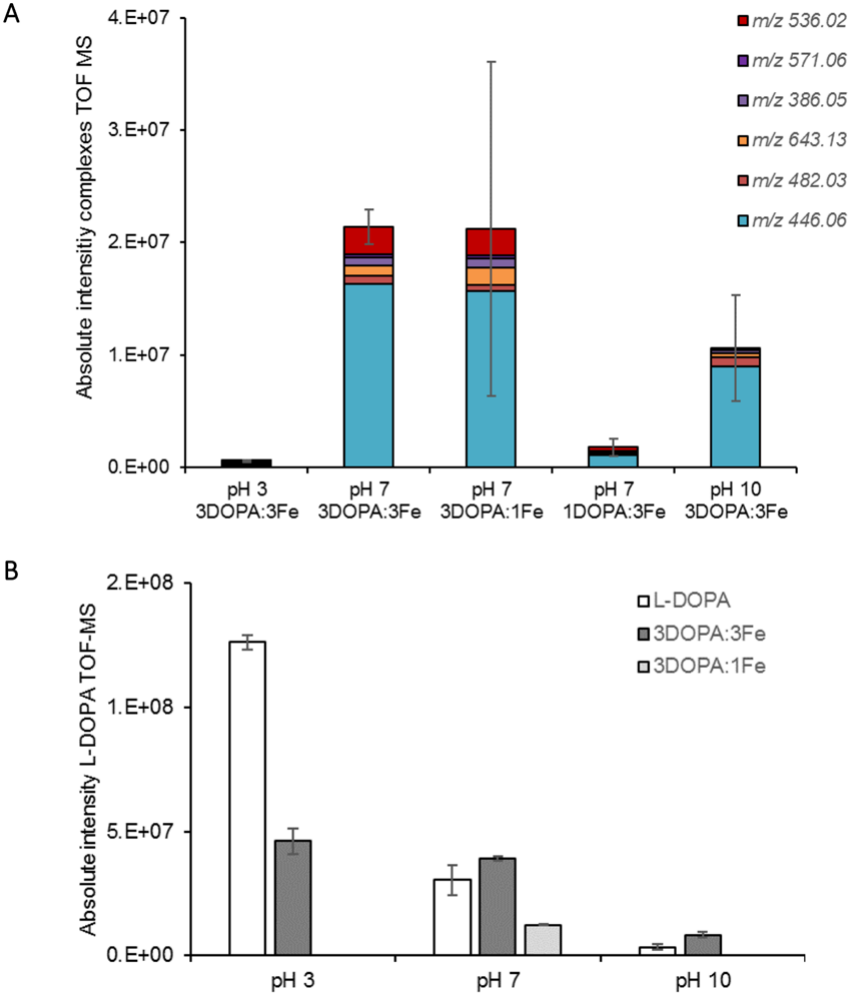
Panel (A) Absolute intensity after direct injection in TOF-MS of different iron-L-DOPA complexes (different *m/z* values) at pH 3, 7 and 10 using equimolar and different ratios of L-DOPA and iron. Panel (B) absolute intensity of L-DOPA without iron and equimolar addition of iron at pH 3, 7 and 10 and ratio 3L-DOPA:1Fe at pH 7 (n = 2, error bars represent absolute deviation).

After identification of the complexes, the complex formation was investigated at different pH and with various L-DOPA: Fe ratios. The total absolute intensity of the different complexes is shown in Fig. 3A for pH 3, 7 and 10 at equimolar ratio and at pH 7 with three different ratios of L-DOPA: Fe.

Few complexes were formed at pH 3 as shown in Fig. 3A, whereas they were formed at higher pH in line with the colour formation (Fig. 2). No mono-complexes (one phenolic combined to iron) were found at low pH, as has been suggested in literature before^18^. Protons were more favourite than iron to bind to L-DOPA hydroxyl groups at low pH^20^. Despite the large variation, complexes at pH 7 and 10 showed comparable intensity in Fig. 3A. In literature, it was reported that the concentration of tris-complexes (three phenolics coordinated by one central iron) were increased at higher pH^17,28^. In this research, pH 7 showed the highest intensity of tris-complexes (*m/z* 643.13), as well as in total intensity of the complexes. At pH 10, part of the variation was likely due to the high reactivity of phenolics at this pH caused by its decreased redox potential^29^.

Formation of L-DOPA-iron complexes at pH 7 was increased by a higher concentration of L-DOPA added, as the summarized signal intensity with 1 mM DOPA:3 mM Fe was lower than that with 3 mM L-DOPA: 3 mM Fe (Fig. 3A). This was in line with the decreased darkening as observed before. The total complex formation with 3 mM L-DOPA: 1 mM Fe (with high standard deviation) was similar to that with 3 mM L-DOPA: 3 mM Fe.

Figure 3B shows the effect of different L-DOPA: Fe ratios on the remaining L-DOPA concentration. L-DOPA without iron was assumed to be stable at pH 3, whereas at pH 10 the amount of L-DOPA decreased, likely due to auto-oxidation. Addition of iron to L-DOPA at pH 3 decreased L-DOPA, due to the redox reaction. Moreover, iron also likely prevented part of the L-DOPA oxidation compared to L-DOPA without iron at pH 7. This prevention of L-DOPA oxidation after iron addition was also found at pH 10, indicating that complexation could partially prevent L-DOPA auto-oxidation (Fig. 3B). This was also described by Xu *et al*.^30^.

### Pattern of oxidation and complexation of diphenolic structures and iron in relation to colour formation in insects

**Figure 4:**
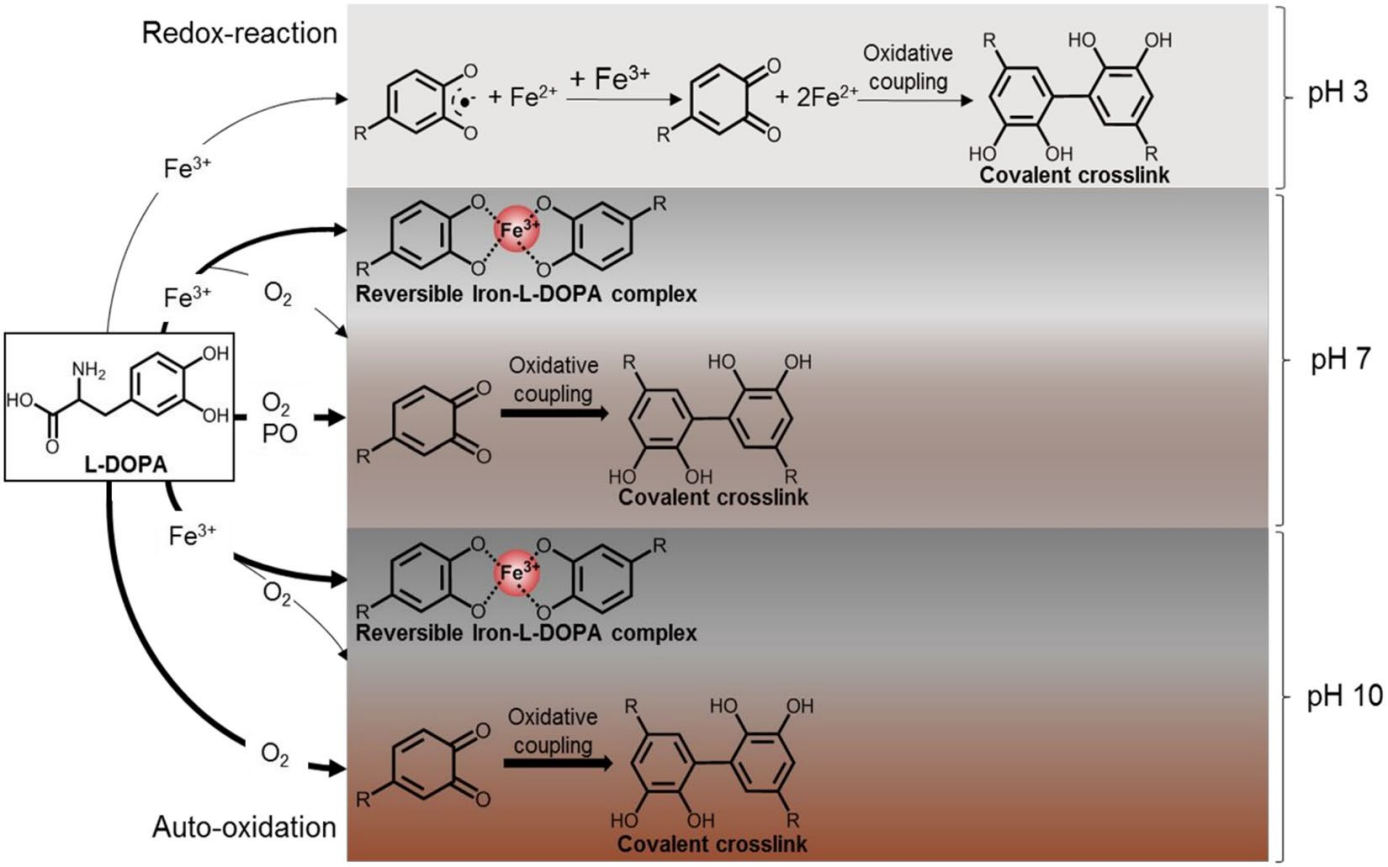
Schematic representation of interactions and reactions between 3 mM L-DOPA and 3 mM Fe^3+^ at different conditions. The thickness of the arrows indicates the extent of the reaction at its pH. R = CH_2_CHNH_2_COOH. Colour shading behind the structures is indicative for the colour shade and extent of colour formation after reaction.

Our results suggested that reversible complexation of L-DOPA by iron and irreversible L-DOPA oxidation were two independent parallel mechanisms as shown for the model system. Factors such as pH and ratio L-DOPA to iron were important determinants in complexation or oxidation. Oxidized phenolics are reactive and can covalently crosslink by e.g. oxidative coupling, forming larger coloured structures that can precipitate. Figure 4 proposes an overall scheme about the fate of L-DOPA under different conditions.

Figure 4 shows the effect of pH on reactions with L-DOPA with or without iron or in presence of phenoloxidase. The reactions playing a role are (i) PO oxidation, (ii) redox oxidation involving iron and/or oxygen and (iii) formation of complexes comprising diphenolic structures and iron. All reactions combined lead to quinones with subsequent covalent crosslinking or formation of complexes comprising diphenolic structures and iron.

At pH 3, a light colour was observed and only limited redox reaction occurred between iron and L-DOPA. Phenoloxidases are inactive at this pH and no iron-L-DOPA complexes were formed. At pH 7, a dark colour was observed caused by the formation of iron-L-DOPA complexation as well as oxidation. Limited auto-oxidation of L-DOPA occurred, but more extensive oxidation was observed when phenoloxidases were added at this pH. At pH 10, iron-L-DOPA complexes were formed and auto-oxidation was more prominent at this pH than at pH 7. Auto-oxidation of L-DOPA at pH 7 and 10 was partly prevented by addition of iron. After oxidation, quinones likely participated in oxidative coupling reactions leading to irreversible covalent cross-linking^17^. Dissociation of iron-L-DOPA complexes upon EDTA addition and fragmentation of the complexes suggested the presence L-DOPA in the complex rather than L-DOPA quinone. The higher the iron concentration, the darker the colour that was formed when L-DOPA was in excess. In insects, this trend in off-colour formation was also observed, as excess L-tyrosine (and therewith L-DOPA) was present compared to iron. Extra addition of iron blackened the colour of *T*. *molitor* and *A*. *diaperinus* extracts, similar to the colour in *H*. *illucens*, which had already a four times higher iron content than the other two species. Even though the model system and insect extracts showed the same trend in colour formation, the iron-phenolic complexes were not evidenced in the insect extracts. This might be due to formation of larger structures with e.g. proteins, which are difficult to detect by mass spectrometry. Based on the similarities in colour formation under different conditions between the model system and during grinding of *H*. *illucens* larvae, we postulate that off-colour formation in *H*. *illucens* followed similar mechanisms as in the model system.

This is the first time that a link between off-colour formation and the presence of iron in insects was made. Iron complexation with phenolic structures and off-colour formation was found before in, for example, iron fortified foods^8^. By identifying colour formation mechanisms in the model system, insight was obtained in the mechanisms playing a role in off-colour formation when processing insects. This understanding in colour formation can help in designing future application of insects for food and feed.

## Materials and Methods

### Materials

*Hermetia illucens* larvae were kindly provided by the Laboratory of Entomology (Wageningen University, The Netherlands). *H*. *illucens* larvae were washed and dried before freezing. *Tenebrio molitor* larvae were purchased from Insectenkwekerij Van de Ven (Deurne, The Netherlands). *Alphitobius diaperinus* larvae were purchased from Kreca Ento-Feed BV (Ermelo, The Netherlands). Larvae were frozen with liquid nitrogen and stored at −22 °C.

Ultra-high-performance liquid chromatography/mass spectrometry (UHPLC-MS) grade formic acid, methanol, hexane, acetonitrile (ACN) and water were purchased from Biosolve BV (Valkenswaard, The Netherlands). Ethylenediaminetetraacetic acid (EDTA) was purchased from Arcros organics (New Jersey, USA).

All other chemicals used were purchased from Sigma Aldrich (St. Louis, USA) or Merck Millipore (Billerica, USA). Water was acquired using a Milli-Q water purification system (Millipore, Billerica, USA). Ferric iron was used as FeCl_3_ (purity ≥ 99.0%), unless stated otherwise. Tyrosinase originated from *Agaricus bisporus*, which was purified to 3000 U/mL as described before^31^.

## Methods

### Colour assessment of insect extracts under different conditions

The larvae of *T*. *molitor*, *A*. *diaperinus* and *H*. *illucens* were blended in MilliQ with 0.1% (w/w) formic acid for pH 3 or 100 mM bicarbonate buffer for pH 7 and 10. Different conditions were tested at pH 7 by adding 1 mM FeCl_3_, 24 mM EDTA or sodium bisulphite (2 g/L)^4^ directly during blending. After centrifugation for 5 min at 12,500 *g*, the colour was assessed by taking pictures in a photo box and/or using spectrophotometric analysis (Shimadzu UV-1800, Kyoto, Japan). The spectrum was measured between 300–800 nm in quartz cuvettes and samples were diluted when necessary.

### Colour assessment of L-DOPA and iron under different conditions

The effect of pH on colour formation was investigated with equimolar concentrations of 3 mM L-DOPA and 3 mM Fe^3+^ in MilliQ with 0.1% (w/w) formic acid for pH 3 or 100 mM bicarbonate buffer for pH 7 and 10. Only at pH 7, different ratios ranging from 1–3 mM L-DOPA and 0.3–30 mM FeCl_3_ were tested. Also for pH 7, an excess of EDTA was added to test the reversibility of colour formation.

The effect of tyrosinase on colour formation was tested at pH 7 using 0.1 M citric acid − 0.2 M phosphate buffer. Tyrosinase from *Agaricus bisporus* was added at 150 units/mL to 3 mM L-DOPA with and without 3 mM FeCl_3_. Colour formation in this solution was monitored for 1 h, and compared to a similar solution without enzyme addition. The colour was assessed by taking pictures in a photo box and / or using spectrophotometric analysis (Shimadzu UV-1800, Kyoto, Japan). The spectrum was measured between 300–800 nm in quartz cuvettes and samples were diluted when necessary.

The effect of oxygen on colour formation was determined by comparing a low-oxygen-level solution with a normal oxygenated L-DOPA solution with ferrous or ferric iron. The low-oxygen concentration was prepared by boiling the MilliQ water used. To confirm the removal of oxygen, the concentration was measured using oxytherm system. Eighty % of the oxygen was removed. DOPA and iron were subsequently solubilized, while keeping the solution under a flow of nitrogen.

### Mineral composition of larvae and insect protein extracts with ICP-AES

Freeze-dried larvae were dried overnight at 70 °C and hydrolysed using concentrated 65% nitric acid and 37% hydrochloric acid by microwave digestion (MARS-X, CEM, USA). Hydrogen peroxide was added to remove the nitrous vapours. The minerals calcium, copper, iron, magnesium, manganese, phosphorus, potassium, sodium, sulphur and zinc were analysed using Inductively Coupled Plasma-Atomic Emission Spectroscopy (ICP-AES) (Varian Vista Pro Radial, Varian Inc., USA), according to the guidelines NPR-6425 and NEN-6966.

### Extraction of polyphenols from insect larvae

A brown and light insect extract for each of the three insects species studied was prepared, in order to investigate the polyphenols changing upon browning. For the light extract, insect larvae were freeze-dried and milled using a 0.5 mm sieve (Ultra centrifugal Mill ZM 200, Retsch, Haan, Germany). Hexane was subsequently used to remove fat. The 10 mg/mL defatted extract was solubilized in aqueous MeOH (50% w/w) with 0.1% (v/v) formic acid. After centrifugation at 12,500 *g* (5 min, at 20 °C), this extract was considered light extract and diluted for analysis.

The brown extract was prepared by grinding (0.5 mm sieve) frozen insects larvae before freeze-drying. The freeze-dried insects were defatted using hexane. The defatted extract was solubilized in water and mixed for 1 h. MeOH with 0.2% (v/v) formic acid was added in ratio 1:1 to stop the browning and obtain the final concentration of 10 mg/mL. After centrifugation at 12,500 *g* (5 min, at 20 °C), this extract was considered brown extract and diluted for analysis.

### Phenolic analysis using RP-UHPLC-UV-MS

Samples were analysed using a Vanquish UHPLC which was equipped with a pump, an auto sampler and a photo-diode array detector (PDA) (Thermo Fischer Scientific, San Jose, CA, USA). Each sample (1 μL) was injected onto an Acquity UHPLC BEH RP18 column (2.1 × 150 mm, particle size 1.7 μm; Waters, Milford, MA, USA), and eluted with UHPLC-MS grade 0.1% v/v formic acid (eluent A) and UHPLC-MS grade acetonitrile, containing 0.1% v/v formic acid (eluent B). The flow rate was 400 μL/min; the temperature of the column oven was 45 °C, with a post column cooler at 40 °C. The sample tray was set at 5 °C. The PDA detector was set to measure over the range of 190–690 nm. The following gradient was used: 0–1.1 min, isocratic on 99% v/v A; 1.1–37.3 min, linear gradient from 1% to 99% v/v B; 37.3–42.8 min, isocratic on 99% v/v B; 42.8–43.9 min, linear gradient from 99% to 1% v/v B; 43.9–50 min, isocratic on 99% v/v A.

Mass spectrometric data were obtained by analysing samples on Q-executive Focus Hybrid Quadrupole-Orbitrap Mass spectrometer (Thermo Fischer Scientific, San Jose, CA, USA), coupled to the UHPLC system. The source voltage was 2.5 kV in the negative ion mode and 4 kV in the positive ion mode. The temperature of the ion transfer tube was 250 °C. Data were collected over the *m/z* range 150–1,500. MS/MS analysis was done on a light or brown mixture of the three species.

Control of the instrument and analysis of the data were done using Xcalibur 2.2 (Thermo Scientific, USA). Analysis was done based on the comparison of light and brown extracts from the three species using compound discoverer v2.1 (Thermo Fisher Scientific, San Jose, CA, USA). To investigate the key components in browning, light and brown extracts were compared within the same species. The phenolics were taken into account when their intensity in the brown extract compared to the light extract was decreased two-fold, and when their intensity in the light extract was above 10^6^. Quantification was done based on standards.

### Sample preparation of complex formation analysis for ESI-Q-TOF-MS

Various parameters (pH, ratio [DOPA]:[Fe] and iron oxidative state), were tested to establish the effect on complex formation. Formic acid in a concentration of 0.1% (w/w) was used for pH 3 and 100 mM ammonium bicarbonate buffer for pH 7 and 10. All experiments were performed with equimolar quantities of L-DOPA and FeCl_3_ (3 mM) unless stated otherwise. A control with EDTA addition in excess to the samples was prepared in order to chelate metal ions present. Each sample was diluted ten times in the buffer at respective pH and centrifuged for 5 min at 12,500 *g* and 15 °C.

### Complex formation and dopachrome formation by Electron Spray Ionization Time of Flight Mass Spectrometry (ESI-Q-TOF-MS)

The samples prepared as previously explained were tested by ESI-Q-TOF-MS to examine the structure of the molecules and complexes responsible for the colour formation. The samples were introduced by direct infusion (μL/min) on Synapt G2-Si high definition mass spectrometer, equipped with a z-spray electrospray ionization (ESI) source, a hybrid quadrupole and an orthogonal time-of-flight (Q-TOF) (Waters, Milford, USA). The intensity of the peaks corresponding to possible iron complexes was annotated in negative mode (NI). Peaks corresponding to dopachrome were annotated in positive mode (PI). L-DOPA was annotated both in PI and NI. The capillary voltage was set to 3.0 kV and 1.8 kV with the source respectively in PI and NI. The source temperature was 150 °C and the sample cone was operated at 30 V in PI and 40 V in NI. MS and MS/MS were performed between *m/z* 25–800 with a 0.3 s scan time and the data was collected for 2 min. The trap collision energy was set at 6 V in single MS mode and optimized in the range between 20 to 30 V in MS/MS mode. Data were acquired and analysed by MassLynx v4.1 (Waters, Milford, USA).

### Significance

Significance between treatments and species were statistically evaluated using t-test with the SPSS 23 program.

## Supplementary information

Supplementary information

## References

[1] Alexandratos, N. & Bruinsma, J. *World agriculture: towards 2015*/*2030: the 2012 revision* (2012).

[2] Rumpold BA, Schlüter OK (2013). Nutritional composition and safety aspects of edible insects. Mol. Nutr. Food Res..

[3] van Huis, A. *et al*. *Edible insects*. *Future prospects for food and feed security*. *Food and Agriculture Organization of the United Nations* **171**, (2013).

[4] Janssen, R. H. *et al*. Involvement of phenoloxidase in browning during grinding of Tenebrio molitor larvae. *Plos One* **12** (2017). 10.1371/journal.pone.0189685 PMC5731683 29244828

[5] Bukkens SGF (1997). The nutritional value of edible insects. Ecol. Food Nutr..

[6] FAO & WHO. Human vitamin and mineral requirements. *Hum*. *Vitam*. *Miner*. *Requir*. 303, 10.1016/B978-0-323-06619-8.10013-1 (2001).

[7] Mellican RI, Li J, Mehansho H, Nielsen SS (2003). The role of iron and the factors affecting off-color development of polyphenols. J. Agric. Food Chem..

[8] Habeych E, van Kogelenberg V, Sagalowicz L, Michel M, Galaffu N (2016). Strategies to limit colour changes when fortifying food products with iron. Food Res. Int..

[9] Nichol H, Law JH, Winzerling JJ (2002). Iron metabolism in insects. Annu. Rev. Entomol..

[10] Andersen, S. O. *Insect molecular biology and biochemistry: cuticlular sclerotization and tanning*. (Elsevier B.V.). 10.1016/B978-0-12-384747-8.10006-6 (2012).

[11] Sugumaran M (2002). Comparative biochemistry of eumelanogenesis and the protective roles of phenoloxidase and melanin in insects. Pigment Cell Res..

[12] Clark KD, Strand MR (2013). Hemolymph melanization in the silkmoth Bombyx mori involves formation of a high molecular mass complex that metabolizes tyrosin. J. Biol. Chem..

[13] Perron NR, Brumaghim JL (2009). A review of the antioxidant mechanisms of polyphenol compounds related to iron binding. Cell Biochem. Biophys..

[14] Bittner S (2006). When quinones meet amino acids: chemical, physical and biological consequences. Amino Acids.

[15] Mira L (2002). Interactions of flavonoids with iron and copper ions: a mechanism for their antioxidant activity. Free Radic. Res..

[16] Hight LM, Wilker JJ (2007). Synergistic effects of metals and oxidants in the curing of marine mussel adhesive. J. Mater. Sci..

[17] Yang J, Cohen Stuart MA, Kamperman M (2014). Jack of all trades: versatile catechol crosslinking mechanisms. Chem. Soc. Rev..

[18] Holten-Andersen N (2011). pH-induced metal-ligand cross-links inspired by mussel yield self-healing polymer networks with near-covalent elastic moduli. Proc. Natl. Acad. Sci. USA.

[19] Kuijpers TFM, Gruppen H, Sforza S, Van Berkel WJH, Vincken JP (2013). The antibrowning agent sulfite inactivates Agaricus bisporus tyrosinase through covalent modification of the copper-B site. FEBS J..

[20] El Hajji H, Nkhili E, Tomao V, Dangles O (2006). Interactions of quercetin with iron and copper ions: complexation and autoxidation. Free Radic. Res..

[21] Andjelkovic M (2006). Iron-chelation properties of phenolic acids bearing catechol and galloyl groups. Food Chem..

[22] Welch KD, Davis TZ, Aust SD (2002). Iron autoxidation and free radical generation: effects of buffers, ligands, and chelators. Arch. Biochem. Biophys..

[23] Vissers A (2017). Enzymatic browning in sugar beet leaves (Beta vulgaris L.): influence of caffeic acid derivatives, oxidative coupling, and coupled oxidation. J. Agric. Food Chem..

[24] Wu YT, Chen YC (2006). Determination of calcium in complex samples using functional magnetic beads combined with electrodeless/sheathless electrospray ionization mass spectrometry. Rapid Commun. Mass Spectrom..

[25] Hider, R. C., Liu, Z. D. & Khodr, H. H. In *Methods in Enzymology* **335**, 190–203 (2001). 10.1016/s0076-6879(01)35243-6 11400368

[26] Barreto WJ (2008). Raman, IR, UV-vis and EPR characterization of two copper dioxolene complexes derived from L-dopa and dopamine. Spectrochim. Acta - Part A Mol. Biomol. Spectrosc..

[27] Elhabiri M, Carrër C, Marmolle F, Traboulsi H (2007). Complexation of iron(III) by catecholate-type polyphenols, Inorg. Chimica Acta.

[28] Sever MJ, Weisser JT, Monahan J, Srinivasan S, Wilker JJ (2004). Metal-mediated cross-linking in the generation of a marine-mussel adhesive. Angew. Chemie - Int. Ed..

[29] Krishtalik LI (2003). pH-dependent redox potential: how to use it correctly in the activation energy analysis. Biochim. Biophys. Acta - Bioenerg..

[30] Xu H (2012). Competition between oxidation and coordination in cross-linking of polystyrene copolymer containing catechol groups. ACS Macro Lett..

[31] Kuijpers TFM (2012). Inhibition of enzymatic browning of chlorogenic acid by sulfur- containing compounds. J. Agric. Food Chem..

