# Supplementary material for: Iron-polyphenol complexes cause blackening upon grinding Hermetia illucens (black soldier fly) larvae
Source: Sci Rep. 2019 Feb 27;9:2967. doi: 10.1038/s41598-019-38923-x (PMC6393531; doi:10.1038/s41598-019-38923-x)
Supplement: Supplementary file 1 — Supplementary information [file 41598_2019_38923_MOESM1_ESM.pdf]

Supplementary information:

Iron-polyphenol complexes cause blackening upon grinding *Hermetia illucens* (black soldier fly) larvae

Renske H. Janssen[1,2], Greta Canelli[2], Mark G. Sanders[2], Edwin J. Bakx[2], Catriona M.M. Lakemond[1], Vincenzo Fogliano[1], Jean-Paul Vincken[2*]

[1]*Food Quality and Design, Wageningen University and Research, PO Box 17, 6700 AA Wageningen, The Netherlands*

[2]*Laboratory of Food Chemistry, Wageningen University and Research, PO Box 17, 6700 AA Wageningen, The Netherlands*

***corresponding author:** jean-paul.vincken@wur.nl

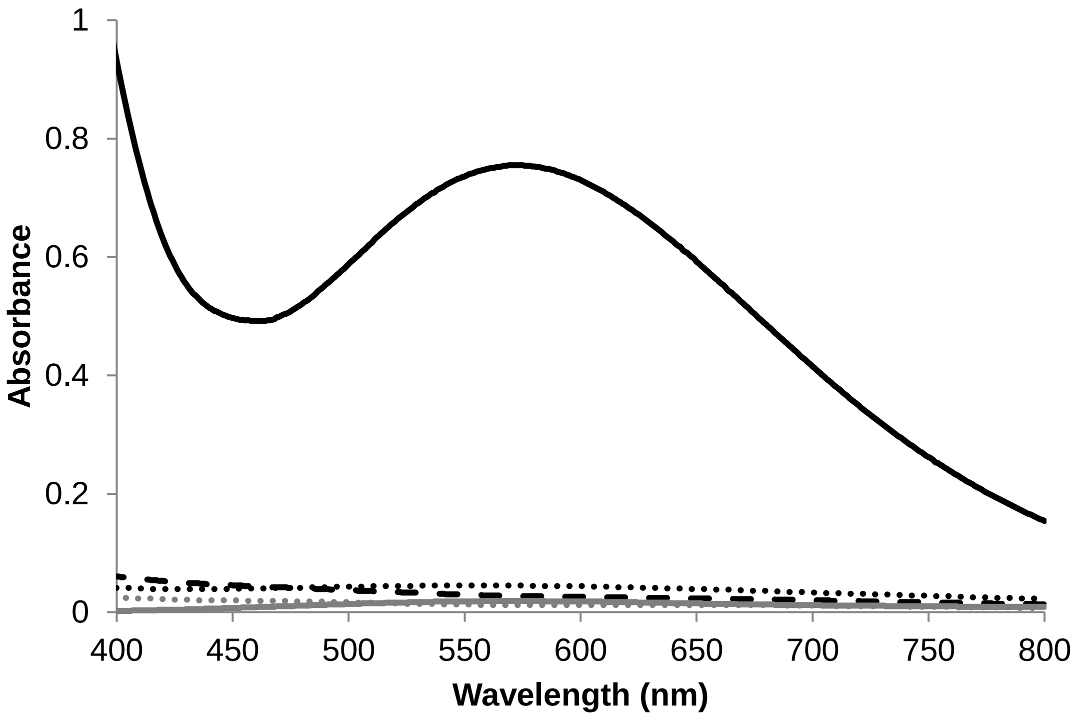

Figure S1. Absorbance of L-DOPA with different minerals: calcium (black striped), manganese (grey dotted), sodium (black dotted), zinc (grey line) and iron (black line). The content of these minerals was significantly different between insect species.

Method:

Effect of minerals (3 mM $CaCl_2$, $MnCl_2$, NaCl, $ZnSO_4$ or $FeCl_2$) was tested at pH 7 using 0.1 M citric acid − 0.2 M phosphate buffer in addition to 3 mM L-DOPA. The colour was assessed using spectrophotometric analysis (Shimadzu UV-1800, Kyoto, Japan). The spectrum was measured between 300-800 nm in quartz cuvettes.

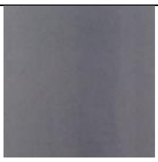	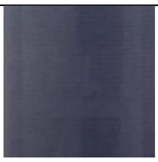	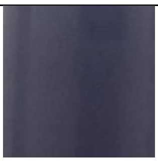	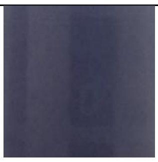
**A**	**B**	**C**	**D**
Ferrous iron	Ferric iron	Ferrous iron	Ferric iron
20% Oxygen	20% oxygen	100% oxygen	100% oxygen

Figure S2. Colour formation in low-oxygen-level L-DOPA solution with ferrous (A) or ferric (B) iron and normally oxygenated L-DOPA solution with ferrous (C) or ferric (D) iron.

Method: Water was boiled to remove the oxygen. To confirm the removal of oxygen, the concentration was measured using oxytherm system. Eighty % of the oxygen was removed. DOPA and iron were subsequently solubilized and kept under nitrogen flow.

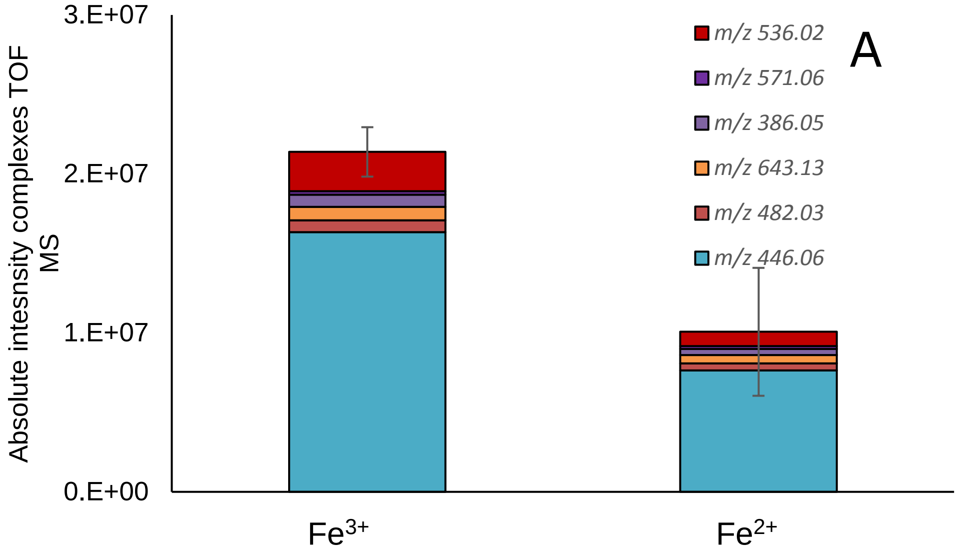

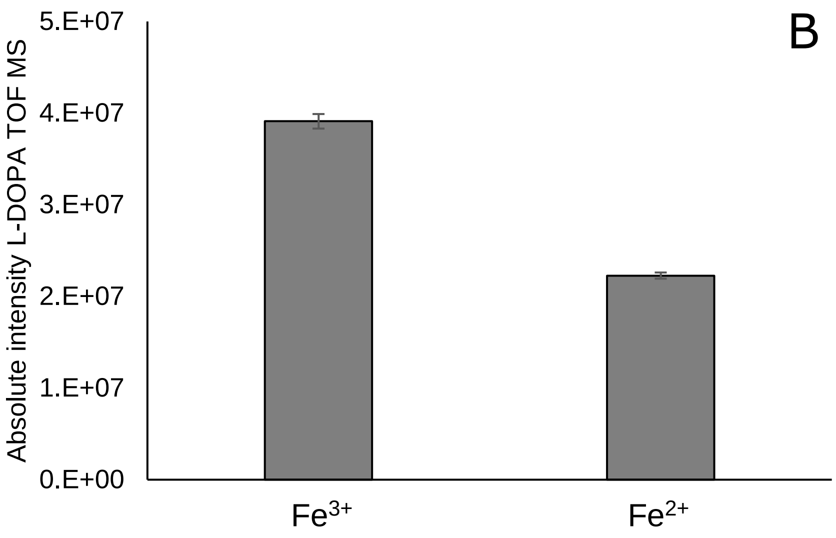

Figure S3. Panel A: Absolute intensity of iron-L-DOPA complexes after addition of 3 mM L-DOPA with 3 mM iron in ferrous or ferric form. Panel B: Absolute intensity of L-DOPA of 3 mM DOPA with 3mM iron ferric or ferrous form (n=2, error bars represent absolute deviation).

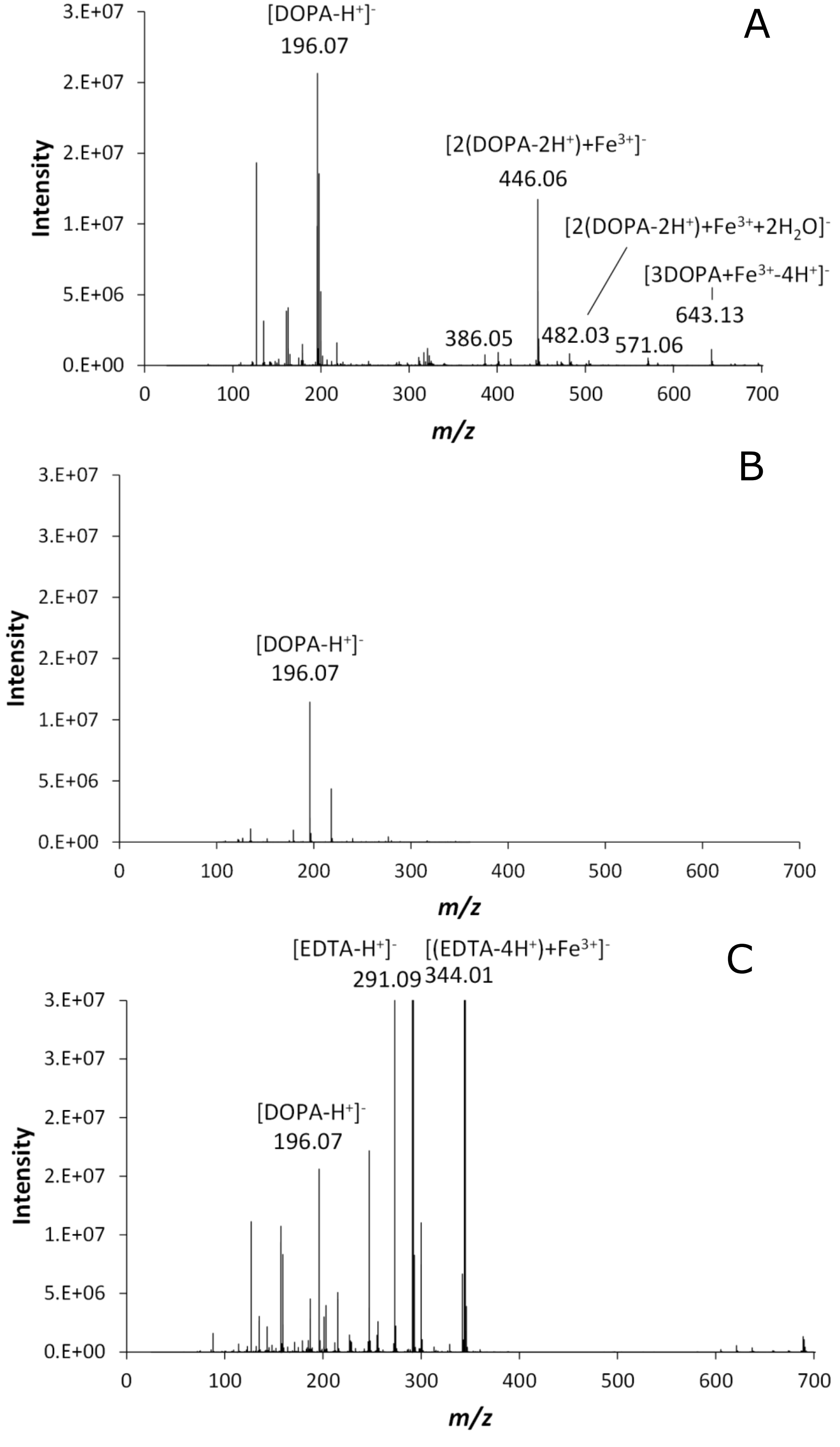
A
$[DOPA-H^+]^-$
196.07
$[2(DOPA-2H^+)+Fe^{3+}]^-$
446.06
$[2(DOPA-2H^+)+Fe^{3+}+2H_2O]^-$
$[3DOPA+Fe^{3+}-4H^+]^-$
386.05
482.03
571.06
643.13
Intensity
m/z
B
$[DOPA-H^+]^-$
196.07
Intensity
m/z
C
$[EDTA-H^+]^-$
291.09
$[(EDTA-4H^+)+Fe^{3+}]^-$
344.01
$[DOPA-H^+]^-$
196.07
Intensity
m/z

Figure S4 Panel A: Electrospray mass spectrum of a solution of L-DOPA (3 mM) + $FeCl_3$ (3 mM) at pH 7 in NI. The peaks at *m/z* 196.07, 446.06, 482.03, 643.13 were identified as $[DOPA\text{-}H^+]^-$, $[2(DOPA\text{-}2H^+)+Fe^{3+}]^-$, $[2(DOPA\text{-}2H^+)+Fe^{3+}+2H_2O]^-$ and $[3DOPA+Fe^{3+}\text{-}4H^+]^-$, respectively. Panel B: Electrospray mass spectrum of a solution of L-DOPA (3 mM) at pH 7 in NI. The peak at *m/z* 196.07 was identified as $[DOPA\text{-}H^+]^-$. Panel C: Zoom of electrospray mass spectrum of a solution of L-DOPA (3 mM) + $FeCl_3$ (3 mM) + EDTA (24 mM) at pH 7 in NI. The peak at *m/z* 273.09, 291.09 and 344.01 showed an intensity of respectively $4.1*10^7$, $4.09*10^8$ and $1.84*10^8$. The peaks at *m/z* 196.07, 291.09 and 344.01 were identified as $[DOPA\text{-}H^+]^-$, $[EDTA\text{-}H^+]^-$ and $[(EDTA\text{-}4H^+)+Fe^{3+}]^-$, respectively.

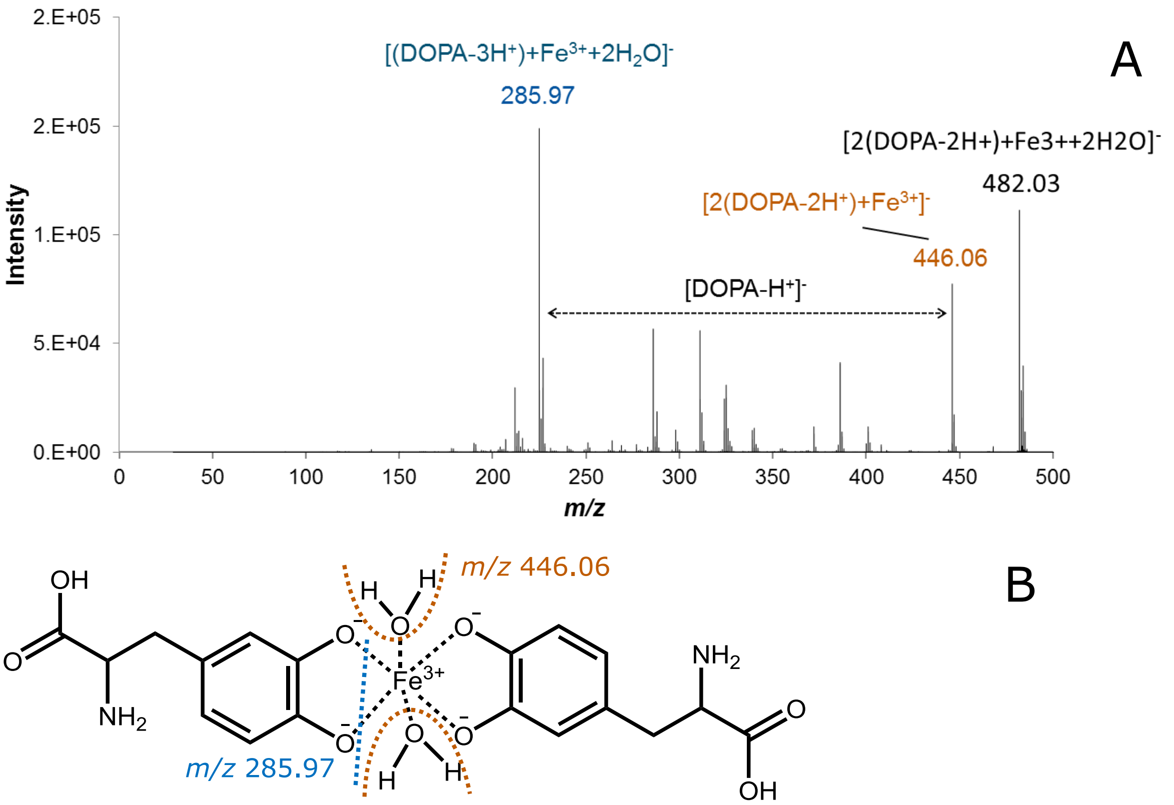

Figure S5 Panel A: MS^2 spectrum of the $[2(DOPA\text{-}2H^+)+Fe^{3+}+2H_2O]^-$. A loss of $[2H_2O]$ (blue) from the parental molecule resulted in daughter ion $[2(DOPA\text{-}2H^+)+Fe^{3+}]^-$ (*m/z* 446.06), whereas loss of $[DOPA\text{-}H^+]^-$ (orange) resulted in daughter ion $[(DOPA\text{-}3H^+)+Fe^{3+}+2H_2O]^-$ (*m/z* 285.97). Panel B: Schematic representation of the main MS^2 fragmentation pattern of $[2(DOPA\text{-}2H^+)+Fe^{3+}+2H_2O]^-$.
